# Supplementary material for: Evaluating a Soil Amendment for Cadmium Mitigation and Enhanced Nutritional Quality in Faba Bean Genotypes: Implications for Food Safety
Source: Plants (Basel). 2025 Jan 6;14(1):141. doi: 10.3390/plants14010141 (PMC11723064; doi:10.3390/plants14010141)
Supplement: Supplementary file 1 [file plants-14-00141-s001.zip › plants-3345741-supplementary.pdf]

Supplementary Information for

Evaluating a Soil Amendment for Cadmium Mitigation and Enhanced Nutritional Quality in Faba Bean Genotypes: Implications for Food Safety

Liping Cheng [1], Jiapan Lian [1,2], Xin Wang [1], Mehr Ahmed Mujtaba Munir [1], Xiwei Huang [1], Zhenli He [3], Chengjian Xu [4], Wenbin Tong [4,*] and Xiaoe Yang [1,*]

[1] Ministry of Education (MOE) Key Laboratory of Environmental Remediation and Ecosystem Health, College of Environmental and Resources Sciences, Zhejiang University, Hangzhou 310058, China
[2] State Key Laboratory for Conservation and Utilization of Subtropical Agri-Biological Resources, Guangxi University, Nanning 530004, China
[3] Department of Soil, Water and Ecosystem Sciences, Indian River Research and Education Center, University of Florida—IFAS, Fort Pierce, FL 34945, USA
[4] Qujiang District Agricultural Technology Extension Center, Quzhou 324022, China
* Correspondence: zjqztwb@163.com (W.T.); xeyang@zju.edu.cn (X.Y.); Tel./Fax: +86-0570-8753680 (W.T.); +86-0571-88982907 (X.Y.)

Contents:

Figure S1. The weather conditions throughout the entire growth period of faba bean

Figure S2. Plant height, ground biomass, Number of pods per plants, Number of seeds per pods, SPAD values and disease score of 11 fava bean genotypes under Control and SA treatments.

Figure S3. Modulations in Elemental Concentrations in Fava Bean Seeds after SA treatment vs. CK.

Figure S4. Phytic acid ratios with Mg, Zn, and Fe.

Table S1. The main physicochemical properties of soil comparing CK and SA treatments were sampled after the harvest of faba beans.

Table S2. Basic Traits of 11 faba bean Genotypes.

Table S3. The fundamental physicochemical characteristics of the tested soil

Text S1. Basic Traits of 11 faba bean Genotypes and Details of Field Experiments

Text S2. Details of Yield and Biomass Analysis in Plant Samples

Text S3. Determination and Analysis of Elements in Plant Samples

Text S4. Determination and Analysis of Phytic Acid Content

Text S5. Determination and Analysis of Soil Collection and Soil Physicochemical Property

Number of pages: 13

Number of figures: 4

Number of tables: 3

Number of texts: 5

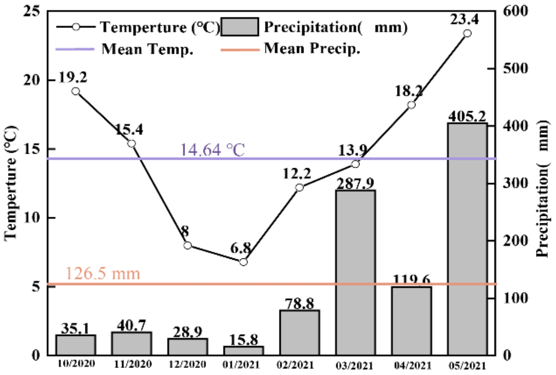

Figure S1. This illustration presents the weather conditions throughout the entire growth period of faba beans, including variations in temperature and precipitation. It also highlights the average temperature and average precipitation during the growth period to provide a more comprehensive assessment of the faba bean growth process.

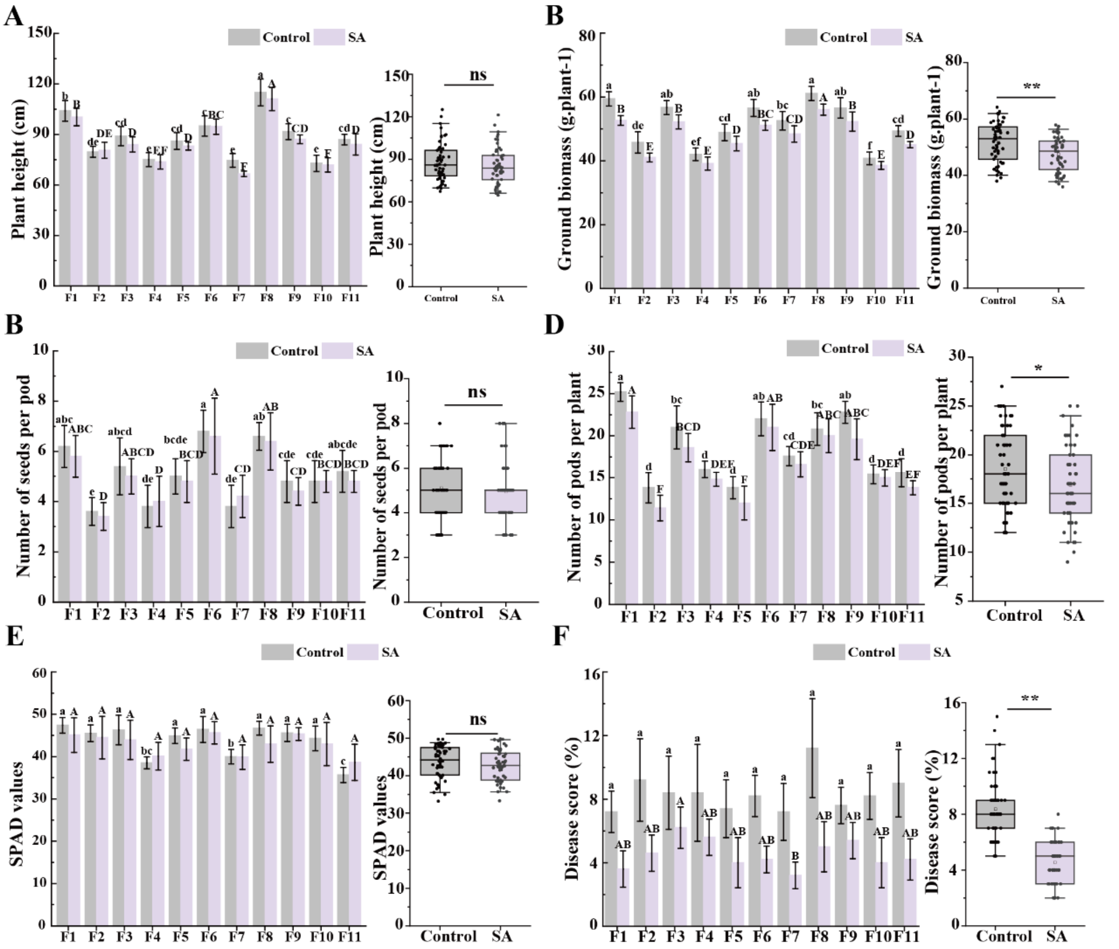

Figure S2. (A) Plant height, (B) ground biomass, (C) Number of pods per plants, (D) Number of seeds per pods, (E) SPAD values and (F) disease score of 11 fava bean genotypes under Control and SA treatments.

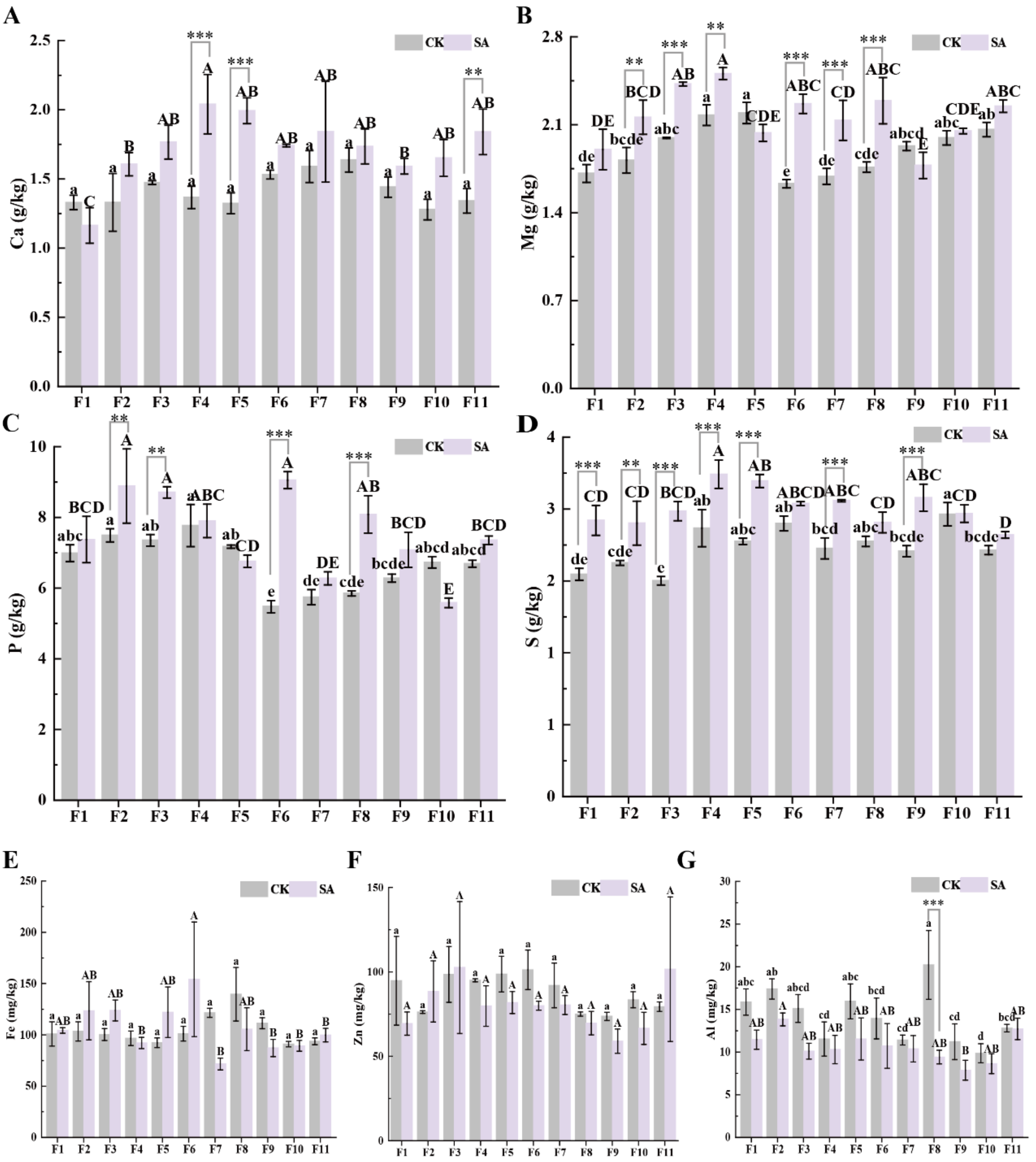

Figure S3. Modulations in Elemental Concentrations in Fava Bean Seeds after SA treatment vs. CK. (A~D) Concentrations of the macronutrients Ca, Mg, P, and S, (E~F) concentrations of the other micronutrient elements Fe, Zn, and Al in seeds, each represented individually.

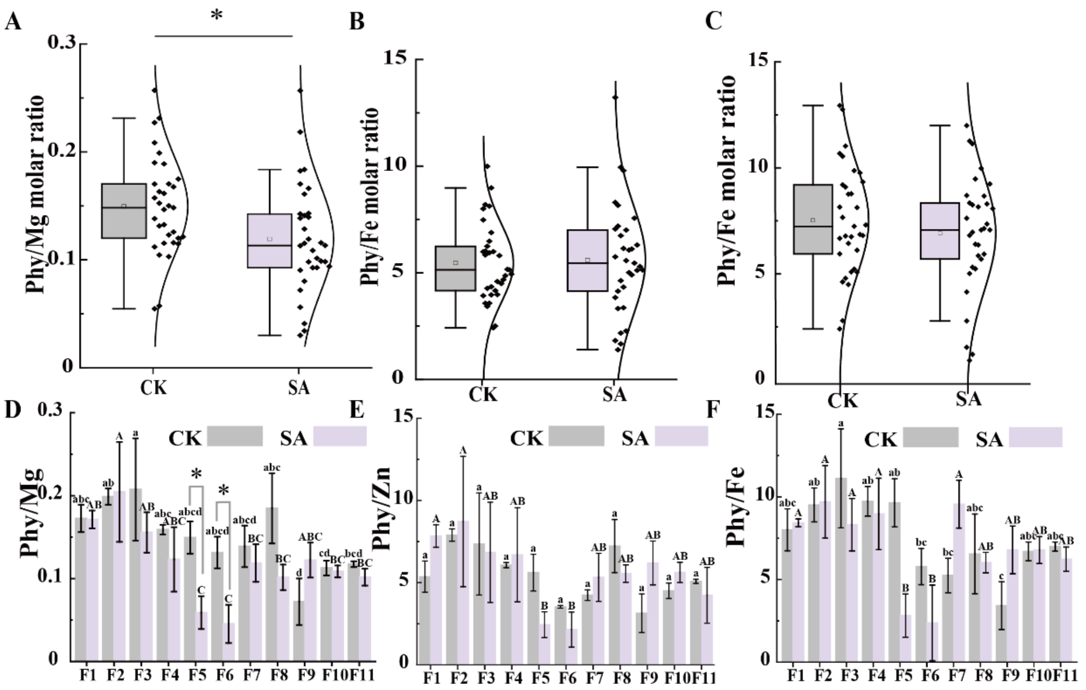

Figure S4. Phytic acid ratios with Mg, Zn, and Fe. (A-C) represent total differences between CK and SA treatments, while (D-F) depict variations among fava bean genotypes under CK and SA treatment.

Table S1. The main physicochemical properties of soil comparing CK and SA treatments were sampled after the harvest of faba beans, with * for $p < 0.05$ and ** for $p < 0.01$ significance levels.

Parameters	Units	Value in CK	Value in SA
pH value	(m_{Soil}:m_{H2O}=1:5)	5.39±0.07	6±0.06
Organic matter	%	20.98±1.4	23±1.69
CEC	Cmol(+)/Kg	14.39±1.02	14.86±0.47
Total N	g/kg	0.71±0.04	0.77±0.03
Total P	g/kg	0.62±0.12	0.67±0.06*
Total K	g/kg	21.94±2.02	22.89±1.41
Total Ca	g/kg	2.02±0.1	3.21±1.56**
Total Cd	mg/kg	1.04±0.02	1.04±0.07
Exchangeable Ca	g/kg	0.37±0.04	0.71±0.28**
DTPA-Cd	mg/kg	0.62±0.02	0.39±0.04*

Table S2. Basic Traits of 11 faba bean Genotypes.

Names	Genotypes	Origin	Seeds	
			Size (cm × cm)	Color
F1	*DFDL*	Jiangsu	2.28 × 1.79	Light green
F2	*DFQL*	Jiangsu	1.92 × 1.41	Green
F3	*RBBH*	Japan	2.43 × 1.80	Light yellow
F4	*QLXF*	Sichuan	2.32 × 1.76	Green
F5	*BDQX*	Shandong	1.90× 1.38	Brown
F6	*NJCD*	Shandong	1.58 × 1.2	Green
F7	*NJHC*	Shandong	1.62 × 1.20	Black
F8	*JHCD*	Shanxi	1.87 × 1.39	Brown
F9	*WMCX*	Henan	2.01 × 1.59	Green
F10	*LCEH*	Hebei	1.76 × 1.43	Brown
F11	*BHDL*	Zhejiang	2.33 × 1.75	Brown

Table S3. The fundamental physicochemical characteristics of the soil in the entire experimental area sampled ahead of the application of any basal fertilizers or soil amendment.

Parameters	Units	Value
Soil Texture	/	Clay Loam
Sand	%	25.03
Silt	%	53.07
Clay	%	21.9
pH value	(m_{Soil}:m_{H2O}=1:5)	5.42 ± 0.06
Organic matter	%	22.65 ± 1.58
CEC	Cmol(+)/Kg	14.87 ± 1.76
Total N	g/kg	0.71 ± 0.03
Total P	g/kg	0.91 ± 0.02
Total K	g/kg	20.4 ± 1.06
Total Ca	g/kg	3.05 ± 0.93
Total Cd	mg/kg	1.3 ± 0.06
Exchangeable Ca	g/kg	0.56 ± 0.16
DTPA-extracted Cd	mg/kg	0.7 ± 0.02

Text S1. Basic Traits of 11 faba bean Genotypes and Details of Field Experiments

The field experiment was conducted on Cd-contaminated agricultural land located in Lijin Garden Village, Quzhou City, Zhejiang Province, China (28.9735° N, 118.8706° E). This region is characterized by a subtropical humid climate, with an annual relative humidity ranging from 70 to 80%. The faba bean cultivation period spanned from October 2020 to May 2021. During this timeframe, the average annual temperature was 14.64°C and the total annual precipitation was 126.5 mm, with detailed monthly climate conditions provided in **Figure S1.** The predominant agricultural practice in the area is the faba bean-rice rotation system, and the soil is predominantly acidic. The fundamental physicochemical properties of the experimental soil include a 5.42 ± 0.06 pH, total Ca of 3.05 ± 0.93 g/kg, available Ca of 0.56 ± 0.16 g/kg, total Cd of 1.3 ± 0.06 mg/kg, available Cd of 0.7 ± 0.02 mg/kg, organic matter of 22.65 ± 1.58 g/kg, total potassium of 0.91 ± 0.02 g/kg, total P of 20.4 ± 1.06 g/kg, and total nitrogen of 0.71 ± 0.03 g/kg (**Table S2)**. According to the China Soil Environmental Quality Risk Control Standard (GB 15618-2018), the Cd risk levels for paddy fields are 0.3 mg/kg (pH ≤ 5.5) and 0.4 mg/kg (5.5 < pH ≤ 6.5), with regulatory limits set at 1.5 and 2.0 mg/kg, respectively. Based on agricultural soil pollution standards and Ca deficiency criteria, the experimental fields were classified as moderately Cd-polluted within regulatory limits, posing potential risks to crops and ecosystems despite an

adequate Ca supply.

The field experiment was conducted using a randomized complete block design (RCBD) with three replicates. Each plot covered an area of 21.6 m2 (1.8 m × 12 m), totaling 66 plots. Before planting the faba beans, all plots received a basic fertilizer application of 30 kg/ha (N: P_2O_5: K_2O= 18:5:27, Purchased from Stanley Ltd.). The SA was **Greenlife No. 1**, formulated by Zhejiang Greenlife Co., Ltd., with its composition detailed in a previous study (X. Wang et al., 2024). In summary, the SA had a pH of 10.5, contained more than 30% CaO, over 15% organic matter, 0.04 mg/kg Cd, 1.84 g/kg sulfur, and, except for manganese at 42.88 mg/kg and iron at 26.67 mg/kg, all other mineral elements were below 2.4 mg/kg. The experimental field was divided into control (CK) and experimental (SA) groups. The experimental group received 6000 kg/ha of **Greenlife No. 1** soil amendment (pH = 11, Ca content ≥ 28%, organic matter content ≥ 14%), which was freshly prepared and incorporated into the soil using a cultivator and labeled as a SA treatment. Before planting, markers were placed to record their positions. During the growing period, in addition to our treatments, the water and nutrient management practices followed those of local farmers.

Text S2. Details of Yield and Biomass Analysis in Plant Samples

Faba beans of all genotypes in the moderately Cd-contaminated farmland,

including both the control (CK) and treatment (SA), were grown in the field for 220 d (October 2020-May 2021). The faba beans were harvested from the central area of each plot (1 m^2/plot) and returned to the laboratory for a subsequent estimate of the theoretical seed yield. Correspondingly, we calculated the disease rate of the faba beans by randomly selecting 100 plants from each sample area and counting the percentages of wilted and decayed plants. Additionally, 10 representative plants were selected from each plot, and the preliminary soil was removed from the roots. After air drying, the plants were returned to the laboratory for classification and analysis. After measuring and recording the plant height, the whole plant was divided into five parts: seeds, pods, stems, leaves, and roots. After washing, all tissues were first dried at 105°C for 30 min, followed by further drying at 65°C in an oven for 48 h. The dry weights of all tissues were recorded and related theoretical agronomic indices were calculated, including the plant dry weight, number of pods per plant, number of seeds per pod, hundred-seed weight, dry biomass, and harvest index (HI). To further analyze the mineral content of different tissues and the nutritional composition of seeds, the dried tissues were ground into fine powders using a high-throughput grinder (Jxfstprp-24, Jinxin, China), packed into No. 5 self-sealing bags, and stored in a 4°C refrigerator for subsequent analysis.

Text S3. Determination and Analysis of Elements in Plant Samples

For elemental analysis, 5 mL HNO3/HClO4 (5:1, v/v) was added to a digestion tube containing 0.2000 g dried powder and the mixture was heated on a graphite furnace preheated to 150 °C for 5 h. The digested solutions from different tissues of faba bean were analyzed for the contents of macronutrients like Ca, Mg, P and S using ICP-OES (ICP6000, Thermo Fisher Scientific, UK), while the concentrations of trace elements such as Zn, Fe, Mo, Mn, Co, Cu, Al and Cd were determined by ICP-MS (Plasma Quant MS, Analytik Jena, Germany). Before the determination, dilute the digestive solution to 50 mL and filtered through a 0.22 μm membrane. Each digestion batch contains blank control, sample duplicates, and standard reference rice flour (SRM 1568a, NIST, Gaithersburg, MD, USA) to check the accuracy of the digestion process. The reference substances were digested in a manner consistent with those used for the samples. To ensure the reliability of the experimental data, the recoveries of the added standards were controlled to between 90-110%.

Text S4. Determination and Analysis of Phytic Acid Content

Twenty mL HCl-Na_2SO_4 mixed solution (1.2%:10%, m/m) was added to a 50 mL plastic centrifuge tube containing 1.0 g of the above seed powder, vortexed to mix thoroughly, and then shaken in a light-proof shaking chamber for 2 h (200 rpm, 25 ℃), and then centrifuged for 15 min (5000g,

25 °C) to collect the supernatant. The supernatant was collected after centrifugation for 15 min (5000 g, 25 °C). An equal volume of the supernatant was mixed with 15% trichloroacetic acid (TCA) extract, vortexed to mix thoroughly, and then left to stand in a refrigerator at 4°C for 2 h. The supernatant was centrifuged at high speed for 15 min, and the pH = 6.25 ± 0.25 was adjusted (drop by drop by adding 0.75M NaOH solution). Sequentially mix the mixed acidity of the clear solution, deionized water and 0.3% sulfosalicylic acid - 0.03% ferric chloride solution reaction in the ratio of 0.1: 1.4: 0.5, the mixed reaction solution was left to stand for 20 min to obtain a blue clarified solution to be measured. The phytic acid concentration of the test solution was calculated from the phytic acid standard curve.

Text S5. Determination and Analysis of Soil Collection and Soil Physicochemical Property

After the faba beans were harvested, three soil samples were collected from both the CK and SA groups using a stainless steel spade, with a sampling depth of 10-20 cm. The soil from both CK and SA was divided into three approximate regions. Within each region, soil was collected from five distinct points using the classic zigzag sampling method. Then, all point samples from each region were thoroughly mixed to obtain a representative composite sample. To avoid contamination and ensure uniformity during the collection and mixing of soils, the process was conducted carefully, resulting

in a total of six composite soil samples. The obtained soil samples were kept air-dried at room temperature. The collected soil was initially milled to remove visible impurities such as stones, weeds, and animal debris, then mixed thoroughly and further milled to a fine powder of uniform particle size. The milled soil was sieved through a 0.15 mm polyvinyl chloride nylon mesh, and the sieved particles were stored in No. 5 sealed plastic bags.

The moderately Cd-contaminated soil was a brown loam, of which 25.03% was clay, 53.07% was silt, and 21.90% was sand. The following basic properties of the soil were determined following the Risk Control Standards for Environmental Quality of Contaminated Soil in Agricultural Soil in China: pH, Organic matter content (g/kg), Total N (g/kg), Total P (g/kg), Total K (g/kg), Total N (mg/kg), Total Cd (mg/kg), Total Ca (mg/kg), DTPA-extracted Cd (mg/kg), DTPA-extracted Ca (mg/kg) (X. Wang et al., 2024).
